# *In silico* screening system based on a transcription factors regulatory network only using transcriptomic data

**DOI:** 10.1371/journal.pone.0319971

**Published:** 2025-04-07

**Authors:** Tadaaki Nakajima, Kentaro Harada, Yasuhiro Tomooka, Tomomi Sato

**Affiliations:** 1 Department of Science, Yokohama City University, Yokohama, Japan; 2 Department of Biological Science and Technology, Faculty of Industrial Science and Technology, Tokyo University of Science, Tokyo, Japan; 3 Graduate School of Nanobioscience, Yokohama City University, Yokohama, Japan; Laboratoire de Biologie du Développement de Villefranche-sur-Mer, FRANCE

## Abstract

In this study, we developed a method to identify core transcription factors (TFs) involved in differentiation using only comprehensive gene analysis. The theory of *in silico* screening using TFs regulatory network analysis (ISNA) required the following requirements: (1) estimating promoter regions, (2) constructing TFs regulatory network (TRN) relationships using the nucleotide sequence information in the promoters and score matrices derived from TF consensus sequences, and (3) identifying candidate core TFs by determining dissociation constants (K_d_ values) within the relationships of TRN. ISNA demonstrated the ability to predict the core TFs involved in the endothelial-to-mesenchymal transition of human umbilical vein endothelial cell (HUVEC) and the differentiation of human embryonic stem cells into mesodermal cells. Using ISNA, we identified HMGA2 as a novel core TF in uterine epithelium. Notably, HMGA2 expression was predominantly detected in uterine epithelium, where it regulated cell proliferation in response to estrogen. These findings highlight ISNA’s potential to identify core TFs based on transcriptomic data.

## Introduction

Various cellular transcriptional regulation and signaling are intricately interconnected, yet the differentiation steps in development remains remarkably stable and drug responsiveness is consistently maintained. To bridge the long gap between the complexity of transcriptional regulation and the stability of phenotype expression, network regulations have been investigated. Networks with directed graphs, such as neural networks, are known for their robustness [1], where overall changes less significantly than individual inputs. Further, small changes in critical nodes can drastically reorganize the network, producing diverse outputs and facilitating cellular evolution. Gene networks modeled as directed graphs have been structured as some cellular models [2,3], demonstrating that subtle changes in the activity of a few core transcription factors (TFs) within a gene regulatory network can reorganize the networks.

Most current networks are built using circuit-like models, which define relationships between genes and assign weighting factors based on experimental findings. Thus, enormous amount of data for each relationship and weighting factor is required to build such models. Linkage logic theory was developed to identify the core factors in gene networks “without” relying on weighting factors [4,5]. Circuit-like models treat gene networks as computer-like system and fail to reflect the functional roles of the proteins transcribed from each gene. While weighting factors in network structures may be estimable, relationship information is still needed. Single-cell RNA-seq combined with knockout experiments (e.g., using CRISPER) has revealed relationships of gene regulatory networks in cell-cell communication [6]; however, numerous knockout experiments for each gene are needed to construct one network. Efforts in the bioinformatics have enabled estimation of gene regulatory network using binding motifs of TFs and ChIP-seq data for chromatins or TFs [7–12]. However, the estimation of gene regulatory network required an input network structure (direction of TF regulation of in whole gene expression) derived from literature or databases [13], This reliance limits the ability to optimize networks for less-studied cell types and phenotypes where ChIP-seq data for key TFs remains incomplete.

**Fig 1:**
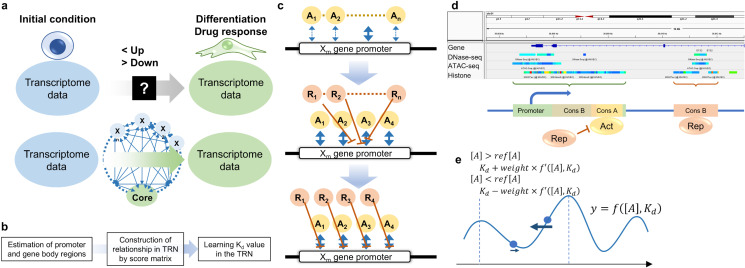
Strategy for development of in silico screening system using a transcription factors regulatory network. (a) Schemes to identify candidate genes and signaling pathways involved in differentiation or pharmacological effects using transcriptome data. In the upper traditional scheme, up- and down-regulated genes are identified but the core signaling pathways remain still a black box. In the lower scheme established in this study, relationships among each gene were predicted using only comprehensive input data. (b) The requirements to develop the ISNA theory. (c) To estimate all TFs binding to each gene promoter, Sp (reflecting activator binding) and Sq values (reflecting repressor binding) were calculated using score matrixes. The top 4 binding sites were listed in a gene promoter and selected a most competitive TF to each binding site as a repressor. (d) The image of selection for regions of TF-bindable promoters and gene bodies from ChIP-Atlas data and binding sites of activators and repressors. (e) Learning K_d_ values in ISNA using a gradient descent method.

To identify of novel candidate genes and signaling pathways involved in differentiation or drug responses in minor cell types and phenotypes, *de novo* comprehensive gene analysis like RNA-seq has been used. While comprehensive gene expression data provides a snapshot at the initial and final states, the intermediate signaling pathways remain unclear (Fig 1a, upper scheme). In developmental biology and pharmacology, even a rough estimation of a core network system “without” predefined weighting factors and input networks can be highly valuable. For example, Manual construction with a simple network structure in human umbilical cord vein endothelial cell (HUVEC) has provided a detailed view of the endothelial-to-mesenchymal transition (EndMT) phenotype [14]. Such network systems are effective for *in silico* screening to identify candidate core factors involved in differentiation and drug response. However, constructing these networks requires numerous relationships and there is a risk of overlooking important signaling pathways.

We hypothesized that predicting gene relationships using “only” *de novo* comprehensive input data could achieve a simple *in silico* screening system to identify the core factors for differentiation and pharmacological effects with a network structure reflecting the protein functions (Fig 1a, lower scheme). In a snapshot, gene expression can be conceptualized as a TF network, where TFs regulate their own and other TFs’ gene expression, while other signaling via cell-cell communication can be ignored. Transcription is thermodynamically assumed as an amount of mRNA synthesis driven by an amount of RNA polymerase II (Pol II) on the transcription start site (TSS) induced by TFs binding [15]. Estimating the dissociation constant values (K_d_) of all TFs in all TF genes using only comprehensive input data could build an *in silico* screening using a TFs regulatory network analysis (ISNA) system that reflects TF binding “without” requiring for predefined relationships and weighting factors. In this study, we developed the ISNA theory and set 3 key requirements: (1) estimation of promoter regions and TFs-binding gene body regions, (2) construction of TF regulatory network (TRN) relationships using nucleotide sequence data and score matrices of consensus sequence of TFs, and (3) identification of core TFs by determining K_d_ values from predicted TRN relationships (Fig 1b). We validated ISNA by predicting core TFs in HUVEC and human and mouse embryonic stem cell (ESC) and used it to identify novel core TFs in the uterine epithelium.

## Materials and methods

### Network prediction

Active and repressive TRNs were automatically predicted using $A\left(x_{m,n}\right)$ and $R\left(x_{m,n}\right)$ which are output as csv files (Act_ or Rep_list.csv) after running of ISNA, respectively. In the analysis from the initial state to the reference state, the digraphs were plotted as ratio of each value (the initial to reference sate) to the control value (the initial to initial state).

### Animals

Female C57BL/6J mice (CLEA Japan, Inc., Tokyo, Japan) for immunohistochemistry and CD-1 mice (Sankyo Research Laboratories Tokyo, Japan) for organ culture were maintained on a 12 h light/12 h dark schedule in a 24 ±  1°C environment. They were given a commercial diet (MF; Oriental Yeast Co., Ltd., Tokyo, Japan) and tap water *ad libitum*. All animals were maintained in accordance with the NIH Guide for the Care and Use of Laboratory Animals, and all experiments were approved by the Institutional Animal Care Committee of Yokohama City University (the approval number: H-A-22-003) and Tokyo University of Science (the approval number: K17007) and performed in accordance with ARRIVE guidelines. All mice were euthanized by cervical dislocation by well-trained individuals. The oviducts, uteri, and vaginae were harvested at postnatal day 3 (P3) and 3 months. The estrous cycle in the intact mice was checked by vaginal smear. C57BL/6J mice at 3 months were ovariectomized (OVX) at 10 days before E2 treatment, injected s.c. with 0.1 μg/25 g BW E2 in sesame oil or vehicle, and harvested at 16 h after the treatment.

### Immunofluorescence

The oviducts, uteri, and vaginae were fixed in 4% paraformaldehyde (PFA) at 4^o^C overnight and dehydrated with graded alcohol. They were embedded in paraffin and cut into 6 μm sections. Sections were deparaffinized with xylene, rehydrated, and rinsed with PBS. For antigen retrieval, sections were immersed in 0.05% sodium citraconic acid (pH 7.4) at 95^o^C for 45 min. Nonspecific binding was blocked in PBS containing 5% goat serum and 0.1% Triton X-100 for 30 min at room temperature. Sections were incubated at 4^o^C overnight with primary antibody for HMGA2 (1/100; Cell Signaling Technology, Danvers, MA, USA) or DLX5 (1/100; Abcam, Cambridge, UK). The sections were treated with Alexa488-conjugated goat anti-mouse IgG (1/300; Thermo Fisher Scientific, Waltham, MA, USA) for 1 h at room temperature. For negative controls, normal rabbit IgG (Santa Cruz Biotechnology, Dallas, TX, USA) was used. 4’, 6-diamino-2-phenylindole (DAPI) was used to stain nucleic acids.

### Organ culture

At 9 weeks, the uteri of OVX CD-1 mice at 7 weeks were collected. Eight volumes of Cellmatrix type I-A (Nitta Gelatin, Osaka, Japan) were mixed with 1 volume of 10 ×  DMEM/F12 and then 1 volume of 200 mM HEPES buffer containing 262 mM NaHCO3 and 0.05 N NaOH was added to the mixture. This 1 mL cold gelatin mixture was poured into cell culture inserts (Corning Inc., Corning, NY, USA) and placed into wells of a 12-well plate, and allowed to gel at 37^o^C for 30 min. The uteri were washed 3 times in HBSS and mixed with fresh 1 mL cold gelatin mixture. The tissues and gelatin mixture were overlaid onto the base of gelled collagen in each cell culture insert and allowed to gel at 37^o^C for 30 min. Subsequently, 1 mL DMEM/F12 with 20% fetal bovine serum (Tissue culture biologicals, Long Beach, CA, USA) with or without 10^-7^ M E2 and 1% 1 mg/mL Hoechst 33258 solution (Dojindo, Kumamoto, Japan, final concentration was 10 μg/mL) were poured in each bottom well. These samples were cultured at 37^o^C in a humidified incubator at 5% CO_2_ for 20 h. To detect cell proliferation, these samples were then cultured in 1 mg/ mL EdU-containing medium for 4 h.

### EdU staining

After incubation with an EdU-containing medium, the samples were fixed in 4% PFA at 4^o^C overnight (n = 5). The samples were embedded in paraffin and cut into 6 μm sections. Sections were stained with Click-iT EdU Alexa Fluor 594 Imaging Kit (Thermo Fisher Scientific), according to the manufacturer’s instructions. The number of EdU-positive cells and DAPI-stained cells was counted by Image J (NIH, Bethesda, MD, USA). Data were expressed as means ±  standard errors. For multiple comparisons, differences were estimated using a Peritz test. A statistically significant difference was defined as *p* ≤ 0.05.

## Results and discussion

### Theory of concentration of TF proteins regulated by TRN in a snapshot

The concentration of TF proteins was modeled within the TRN. In a hypothetical unicellular events in a snapshot, where transcriptional regulation is unaffected by environmental factors, we hypothesized that the TF concentrations were solely regulated by TRN and eventually stabilized based on the initial TF concentrations and interactions of the TRN. To isolate the effects of TRN regulation, post-transcriptional and post-translational modifications was excluded from this model. To estimate transcriptional regulation among TFs, we focused on TFs with known consensus sequences. Specifically, we used 810 human TFs listed in JASPAR2020 (https://jaspar.genereg.net/). The TF concentration at a given step *n* was represented as $x_{m,n}$ ($m\in TFnumber\left(810\right)$, learning step number n∈ℕ) and its change over time was defined as:


$$x_{m,n+1}=f\left(x_{1,n},x_{2,n},\ldots ,x_{810,n}\right)+g\left(x_{m,n}\right)$$ (1)

Here, $f\left(x\right)$ represents regulation of the gene expression within the TRN, while $g\left(x\right)$ is a function for a residual amount by degradation. In this study, proteins in a nucleus assumed to be degraded by proteasomal and autophagic pathways, primarily regulated via ubiquitination by an enzymatic reaction [16]. The degradation function follows Michaelis-Menten kinetics and is expressed as:


$$g\left(x_{m,n}\right)=W\left(e^{-d+logx_{m,n}+x_{m,n}}\right)$$ (2)

where W is Lambert W, and (d∈ℝ) value represents the degradation rate.

### Direct estimation of TF network regulation via by TF binding

The rate of mRNA synthesis can be thermodynamically estimated by RNA polymerase II and TFs binding [15].


$$f\left(x_{1,n},x_{2,n},\ldots ,x_{810,n}\right)=k_{c}a_{g}\text{exp}\left(\frac{-E_{p_{g}}-\Delta G_{p_{g}}^{0}+E_{d_{g}}}{RT}\right)$$ (3)

Here, k_c_ is a constant for basal transcription activity, which varies with RNA polymerase concentration. Temperature is stable in the mammalian cells. A value of a_g_ is a constant for the chromatin state. In this study, we estimated the stable RNA polymerase concentration and temperature, and modeled only opened chromatin; therefore, those values (k_c_, a_g_, and RT) are collectively represented by k_c_. $E_{d_{g}}$, a member for RNA degradation, is omitted for simplification. $\Delta G_{p_{g}}^{0}$, a member for RNA polymerase II binding, and $E_{p_{g}}$, a member for mRNA-synthesizing activity of RNA polymerase II, both are regulated by TFs.


$$f\left(x_{1,n},x_{2,n},\ldots ,x_{810,n}\right)=k_{c}\text{exp}\left(-E_{p_{g}}-\Delta G_{p_{g}}^{0}\right)$$ (4)

For a single activator A with concentration [A] =  x_m,n_, let r represent the number of binding for p of promoter sequences on a gene promoter, which depends on [A] since the concentration [S] of the binding sequence on the genome is stable. When [S] is set as 1 to simplify, r is estimated using the p value which is the number of binding sites.


$$\text{r}=\left[AS\right]=\sum_{p}\frac{\left[S\right]\left[A\right]}{K_{d}+\left[A\right]}=\sum_{p}\frac{\left[A\right]}{K_{d}+\left[A\right]}$$ (5)

When many amounts of one type of activator bind to a target promoter sequence, the activator and sequences can act as a super enhancer, producing a nonlinear and stable effect like computational circuit [17]. We assumed a maximum p of 4 binding sites and additional site’s effects contributed minimally in this theory.

Consensus sequences for activators are expressed as a score matrix. When a binding score Sp value (0 ≤ Sp ≤ 1) was calculated by the score matrix for each consensus sequence increases to 1, the activator binds to the sequences with exponential strength [18]. We introduced the effect of the consensus sequence on the binding sites and a weighting factor “a”


$$\text{r}=\sum_{p}\frac{10^{-a\left(1-Sp\right)}\left[A\right]}{K_{d}+10^{-a\left(1-Sp\right)}\left[A\right]}$$ (6)

To accelerate the calculation of r values, we proposed a list of Sp in all TFs from maximum Sp1 to fourth Sp4 (Fig 1c; see the program “TF_binding”). Further, we considered that other TFs act as repressors for the activator A by antagonistic effects. The strongest antagonist R was select based on a list of sore matrix values for repressors (Sq) and TF concentration using $K_{i}10^{-a\left(1-Sq\right)}\left[R\right]$ (K_i_: dissociation constant of R) (see program “Act_Rep_score_making”).


$$\text{r}=\sum_{p}\frac{10^{-a\left(1-Sp\right)}\left[A\right]}{K_{d}+10^{-a\left(1-Sp\right)}\left[A\right]+10^{-a\left(1-Sq\right)}\left(\frac{K_{d}}{K_{i}}\right)\left[R\right]}$$ (7)

For promoter with high binding affinity (a sum of Sp1 to Sp4 values exceeding a threshold), we assumed that the r value was exponentially calculated because it will act as a super enhancer [17].


$$\text{r}=\frac{\sum_{p}10^{-a\left(1-Sp\right)}\times {\left[A\right]}^{p}}{{K_{d}}^{p}+\sum_{p}10^{-a\left(1-Sp\right)}\times {\left[A\right]}^{p}+\sum_{q}10^{-a\left(1-Sq\right)}\left(\frac{K_{d}}{K_{i}}\right)\left[R\right]}$$ (8)

Since $E_{p_{g}}+\Delta G_{p_{g}}^{0}$ is inversely proportional to r, a value of $f\left(x\right)$ only by an activator A is:


$$f\left(x_{m,n}\right)=A\left(x_{m,n}\right)=k_{c}\left\{\text{exp}\left(-\frac{1}{r}\right)\right\}$$ (9)

When the TF is only listed as a repressor in gene ontology terms, the function is converted to decrease expression. The function of gene regulation by all TFs is calculated by sums of contributions from all activators and repressors:


$$f\left(x_{1,n},x_{2,n},\ldots ,x_{810,n}\right)=\sum_{m=1}^{810}A\left(x_{m,n}\right)$$ (10)

When TFs bound to regions in a gene body, we assumed that TFs acted as a repressor by the reference of basal procaryotic regulation [19]. If the repressor effects by TFs in a gene body are independent of promoter binding, the repressor effects are calculated as the same method, negatively act on activator effects, and are subtracted from the function of all promoter regulation.


$$f\left(x_{1,n},x_{2,n},\ldots ,x_{810,n}\right)=\sum_{m=1}^{810}\left\{A\left(x_{m,n}\right)-R\left(x_{m,n}\right)\right\}$$ (11)

To prevent negative regulation values, we set:


$$f\left(x_{1,n},x_{2,n},\ldots ,x_{810,n}\right)=0\left(f\left(x_{1,n},x_{2,n},\ldots ,x_{810,n}\right)<0\right)$$ (12)

The TF concentration update equation incorporates both activation and degradation:


$$x_{m,n+1}=\sum_{m=1}^{810}\left\{A\left(x_{m,n}\right)-R\left(x_{m,n}\right)\right\}+W\left(e^{-d+logx_{m,n}+x_{m,n}}\right)$$ (13)

To determine optimal K_d_ values for TFs when condition of a cell from initial state with initial transcriptomic data changes to different state with reference transcriptomic data, we applied a gradient descent method. Weighting factors of K_d_ for activator and repressor are set based on reference concentration values from reference transcriptomic data.

### Parameters fitting in ISNA

Using the theory of TF concentration regulated by the TRN, we developed the ISNA algorithm, which includes: (1) estimation of promoter and gene body regions, (2) listing Sp and Sq values using score matrixes, and (3) optimizing K_d_ value in the TRN using reference data of RNA-seq (Fig 1b). To identify open chromatin regions in the genome, we utilized the database of ChIP-Atlas (https://chip-atlas.org/). Open chromatin regions were defined as connected positive regions detected by ChIP-seq for all modified histone, DNase-seq, and ATAC-seq, with at least one positive region from DNase-seq or ATAC-seq (Fig 1d). In heterochromatin-dense regions, gene expression will be constitutively “off” without regulation by TFs. Therefore, such regions were excluded from the analysis unless they exhibited ATAC-seq or DNase-seq signals. H3K27ac, H3K4me3, and H2K4me1 are used for active histone marks and H3K27me3 and H3K9me3 are used for repressive histone marks [20]. Initially, we attempted to use active histone marks to estimate the open chromatin regions; however, in some cases, no open chromatin region was identified in genes although the genes are actively expressed in the cells. We considered that the failure to estimate the open chromatin regions was caused by which studies of effects of histone modification on gene expression are ongoing. For example, repressive histone marks are sometimes detected with ATAC-seq and DNase-seq signals as a silencer region [21]. Thus, we incorporated data from all histone modifications to improve the accuracy of open chromatin region identification.

**Fig 2:**
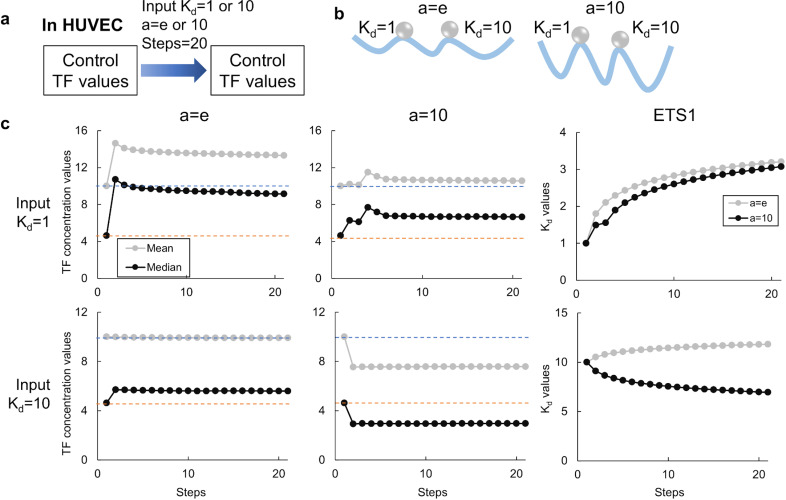
Learning K_d_ values in ISNA. (a) The condition for learning K_d_ values in ISNA. The same HUVEC RNA-seq data was used as the input and reference expression data. One or 10 values were used as input K_d_ values for all TFs. The values of “a” as a weighting factor for effects of Sp and Sq values on binding TFs were set to e or 10. (b) The schematic image of learning K_d_ values. (c) The mean and median TF concentration values in all TFs and the K_d_ value of ETS1 in learning K_d_ values in 20 steps.

In open chromatin regions, those containing transcription start sites were defined as promoter regions, while the regions spanning from transcription start sites to end sites were defined as gene body regions. In HUVEC, the 343 genes of TFs were identified as genes including open promoter regions. After listing Sp and Sq values using score matrixes based on consensus sequences of each TF, the concentration of TFs was set using RNA-seq data from 2 conditions (e.g., undifferentiated vs. differentiated conditions) as initial and reference values. The K_d_ value in TRN was optimized using a gradient descent method (Fig 1e). For parameters fitting “a” which is a weighting factor for the score matrixes and an the initial K_d_ value, we ran the ISNA program using the control HUVEC RNA-seq data [22] as initial and reference values for 20 gradient descent steps (Fig 2a). If TRN stability was visualized as a mountain landscape, a higher weighting factor “a” for score matrixes reflects a steeper slope of Sp and Sq values in TF binding, while different initial K_d_ values correspond to different start points (Fig 2b). When the same values are used to initial values as reference values, the ideal outcome was for ISNA to maintain TF concentrations equal to the initial values (Fig 2c, dashed lines). When the weighting factor “a” was set low (e.g., e) or high (e.g., 10), the K_d_ values were stable at initial or different values. Under any conditions, the K_d_ values usually asymptotically relate to some values and do not fluctuate (e.g., the K_d_ values of ETS1). Therefore, suitable parameters for each analysis can be selected by adjusting the weighting factor for the score matrixes and the initial values of the TFs.

### ISNA identified core TFs related to differentiation in HUVEC and ESC

**Fig 3:**
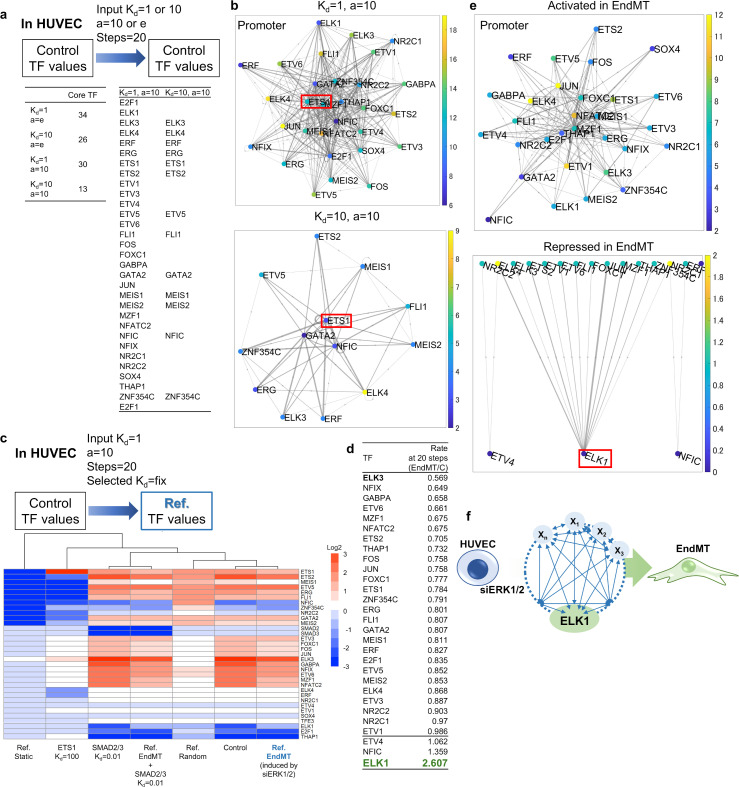
Identification of core TFs in endothelial to mesenchymal transition in HUVEC using ISNA. (a) The condition for learning K_d_ values in ISNA and the table of the number and name of core TFs in which the K_d_ values were changed after learning. (b) The digraph was derived from derivative factors of the gradient descent method to visualize the relationships among core TFs at each promoter region. When the TFs had a larger number of edges with the entering direction to the TFs, the TFs were situated at a more center position. Left bar and color of TFs: the number of edges with the forward direction from the TFs. Width of edges: reflecting the size of derivative factors. (c) Heatmap of log2 fold change of rate of K_d_ values (values after analysis/initial values) for the core TFs in the TRN regulating the gene expression from control HUVEC to reference situation. When SMAD2/3 or ETS1 was selected, the K_d_ values were fixed in the analysis. (d) List of rates of K_d_ value in the TRN regulating the gene expression from control HUVEC to EndMT. (e) The digraph was derived from the rate of derivative factors of the gradient descent method (EndMT/control values) in activated or repressed TFs in EndMT to visualize the relationships among core TFs at each promoter region. (f) The schematic image of TRN and core ELK1 to induce EndMT in HUVEC.

Under specific parameter setting, K_d_ values were optimized using HUVEC RNA-seq data [22] as both initial and reference values for 20 gradient descent steps (Fig 3a). In the HUVEC’s TRN consisting of 343 TFs, many TFs retained the initial K_d_ values after optimization, suggesting that those TFs do not play a regulatory role in the TRN. Thus, we defined candidates of core TFs characterized by different K_d_ values after optimization. While the number of core TFs varied depending on parameter selection, smaller core TF sets were always subsets of larger ones (Fig 3a). The relationships among TRN were visualized using a digraph derived using derivative factors from gradient descent method (Fig 3b in promoter regions; S1 Fig in gene body regions). For example, ISNA identified ETS1 as the candidate of key core TF in HUVEC’s TRN. This is consistent with previous findings that TFs belonging to the ETS family regulate endothelial cell development [23], and that inhibition of ETS1 disrupts endothelial cell barrier function [24].

To model TRN during the transition from control to experimental conditions in ISNA, the phenomenon of EndMT in HUVEC was selected as an example. Using the RNA-seq data of HUVEC in the state of EndMT induced by ERK1/2 knockdown [22] as the reference value, ratio of the group of all K_d_ values after fitting was calculated compared with that using the control HUVEC RNA-seq data in the set of parameters (weighting factor a = 10, initial K_d_ value = 1) (Fig 3c). When continuous activation of SMAD2/3 (known as EndMT inducers and induced by TGFβ ligands, but not selected as a core TF in ISNA) was simulated by setting K_d_ value = 0.01, it had no effect other K_d_ values of TRN components after optimization. On the other hand, continuous inhibition of ETS1 by setting the K_d_ value = 100 drastically altered the other K_d_ values of TRN components. Under these conditions, even using random and constant reference values, only the K_d_ values of core TFs changed from their initial values. By calculating the ratio of K_d_ values of the core TFs in the EndMT group/control group, ELK1 was identified as the most inhibited core TF to induce EndMT (Fig 3d). The differential relationships within the TRN under EndMT vs. control conditions were visualized as a digraph (Fig 3e in promoter regions; S2 Fig in gene body regions). In particular, ISNA predicted that several core TFs were activated and ELK1 was repressed during EndMT condition. Therefore, ISNA identified ELK1 as the most critical core TF for induction of EndMT in HUVEC’s TRN (Fig 3f). This finding aligns with previous studies showing that ELK1 mediates fibroblast growth factor signaling to inhibit EndMT, and that ELK1 knockdown induces EndMT in endothelial cells [25].

**Fig 4:**
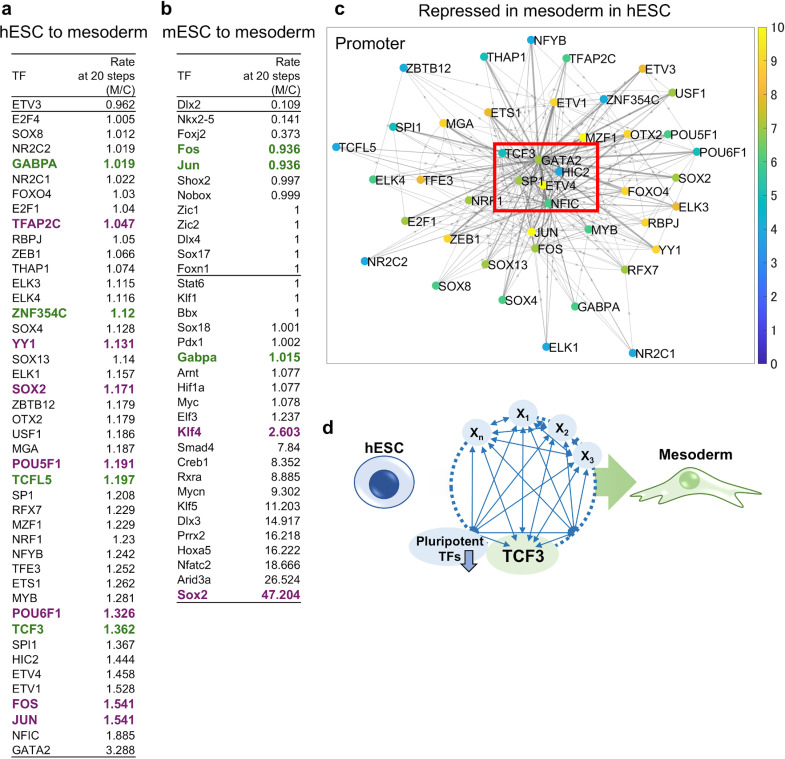
Identification of core TFs for differentiation into mesodermal cells in human and mouse ESC using ISNA. Lists of rates of K_d_ value in the TRN regulating the gene expression from hESC (a) and mESC (b) to mesodermal cells. (c) The digraph was derived from the rate of derivative factors of the gradient descent method (mesodermal cells/hESC values) in repressed TFs in EndMT to visualize the relationships among core TFs at each promoter region. When the TFs had a larger number of edges with the entering direction to the TFs, the TFs were situated at a more center position. Left bar and color of TFs: the number of edges with the forward direction from the TFs. Width of edges: reflecting the size of derivative factors. (d) The schematic image of TRN and core TFs related to pluripotency to induce mesodermal cells in HUVEC.

To further validate ISNA’s ability to model TRN transitions, we analyzed mesodermal differentiation in human ESC [26] and mouse ESC [27]. In hESC, the TRN included 480 TFs out of 810 possible TFs with known consensus sequence, while in mESC, the TRN included 355 TFs from 596 possible TFs. Core TFs were estimated under the parameter set (weighting factor a = 10, initial Kd value = 1). The ratios of K_d_ values of core TFs in the mesodermal differentiation vs. control conditions in human ESC were listed (Fig 4a). ISNA identified the inhibition of many TFs related to pluripotency (e.g., POU5F1 and SOX2) and involvement of TCF3 activated by Wnt signaling. In mESC, fewer TFs were identified as core TFs and important TFs related to pluripotency and mesodermal differentiation are reduced compared to that in hESC (Fig 4b). The lower performance of ISNA for the identification of core TFs in mESC indicated the importance of the accumulation of information on the consensus sequence of TFs and ChIP-seq on chromatin states. The differential relationships among the TRN in the mesodermal differentiation vs. control conditions in hESC were visualized as a digraph (Fig 4c), highlighting the key set of core TFs, including TCF3. Therefore, ISNA identified TCF3 as a critical core TFs for mesodermal differentiation in hESC’s TRN (Fig 4d). This finding aligns with previous research showing that activation of Wnt signaling in ESC induces loss of pluripotency, remodeling of plastic chromatin to differentiate into mesoderm, and mesodermal specification [28,29].

### ISNA identified novel candidates of core TF in uterine epithelial cells

**Fig 5:**
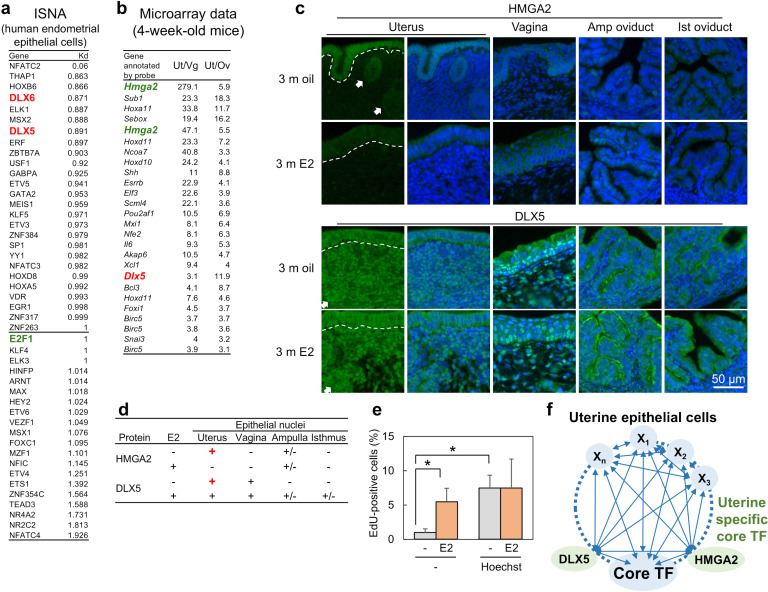
Identification of new specific core TF in uterine epithelial cells using ISNA. (a) Lists of K_d_ value in the TRN regulating the gene expression in human endometrial epithelial cells. (b) The ratio of gene expression between the uterus and vagina or the uterus and oviduct in 4-week-old mice was listed using data examined with microarray. (c) The immunofluorescence images for HMGA2 or DLX5 (green) in the uterus, vagina, ampulla of oviduct, and isthmus of oviduct of 3-month-old OVX mice with oil or E2 treatment. Blue: the nuclei. White arrows: the uterine glands. Dash line: basement membrane. n = 3, biologically independent. (d) The table of expression for HMGA2 and DLX5 in the nuclei of the epithelium of female reproductive tracts in adult mice with or without E2. (e) The percentage of EdU-positive cells in the epithelium of organ-cultured uterus with or without E2 and HMGA2 inhibitor, Hoechst 33258. * : *p* ≤ 0.05. n = 5. (f) The schematic image of TRN and specific TFs in adult uterine epithelial cells.

To identify previously unknown core TFs, we applied ISNA. In the female reproductive tract, the vaginal and oviductal epithelium can be specified by the expression of TFs, TRP63 and FOXJ1, respectively [30,31]; however, no specific TF in uterine epithelial cells has been identified. To construct the TRN in uterine epithelial cells in ISNA, the RNA-seq data from the human endometrial epithelium [32] was used as both the initial and reference values. ISNA identified core TFs in human endometrial epithelial cells (Fig 5a; the digraph in S3 Fig). To further refine these candidates, they were compared with the group of genes highly expressed in the uterus of 4-week-old mice relative to the vagina and oviduct [33] (Fig 5b). From this intersection, HMGA2 (which interacts with E2F1) and DLX5 are identified as candidate uterus-specific core TFs. Immunostaining revealed that HMGA2 was strongly expressed in the nuclei of uterine epithelial cells, with weak expression in the oviduct of ampulla in oil-treated ovariectomized (OVX) 3-month-old mice (Fig 5c, 5d). DLX5 was expressed in the epithelium and stroma of the uterus and vagina in the oil-treated 3-month-old mice. No difference of expression of HMGA2 and DLX5 was not observed among female reproductive tracts in estradiol (E2)-treated, estrus, and diestrus 3-month-old mice and mice at postnatal day 3 (S4 and S5 Figs), suggesting that their expression was regulated by sex hormones. DLX5 and DLX6 are known to be important TFs for the development of uterine glands in epithelial regions [34]. HMGA2 promotes cell proliferation and angiogenesis in uterine leiomyomas [35,36], but its function in the uterine epithelium remains unclear. Since estrogen-regulated cell proliferation is a key function of uterine epithelial cells, we hypothesized that the core TFs in this context would be involved in estrogen-mediated cell proliferation. To investigate this, we treated organ-cultured adult uterus with Hoechst 33258 which binds the minor groove of AT-rich DNA and displaces HMGA proteins from chromatin [37,38], alongside E2 (Fig 5e). E2 and Hoechst 33258 treatment induced cell proliferation in the uterine epithelium at similar level, and Hoechst 33258 did not have a synergistic effect with E2 treatment. Because Hoechst 33258 treatment did not inhibit cell proliferation in the organ-cultured uterus, Hoechst 33258 may inhibit TFs with motif of AT-rich region, including HMGA2. Therefore, HMGA2 may act as a repressor of cell proliferation in the uterine epithelium under static condition, while E2 may promote cell proliferation by downregulation of HMGA2 expression. In addition, high HMGA2 expression was detected in the epithelial junction between the oviduct and uterus (S6 Fig), suggesting HMGA2 may support junctional maintenance by inhibition of epithelial cell proliferation. Thus, the expression of HMGA2 (and to some extent, DLX5) in the epithelium of the female reproductive tracts defines the identity of uterine epithelial cells with the E2-HMGA2 signaling pathway regulating cell proliferation. These findings demonstrate that ISNA can support the identification of tissue-specific core TFs only using transcriptomic data (Fig 5f).

## Conclusion

ISNA enables construction of TRN composed of core TFs, bridging the gap between initial to the reference gene expression states. Notably, ISNA constructs TRN using only transcriptomic data “without” requiring predefined parameters such as directionality, weighting factors among TFs, and an input network structure. However, ISNA’s ability to identify core TFs related to differentiation in mESC is weaker than that in hESC, suggesting that ISNA is dependent on the accumulation of information from consensus sequence of TFs and ChIP-seq for chromatins. Further, ISNA operates under several simplifying assumptions, limiting its applicability. For example, ISNA does not account for cell-cell interactions and post-transcriptional regulation, as these factors were excluded from its functional framework. To learn optimal K_d_ values for TFs, ISNA employs a gradient descent method without testing alternative machine learning approaches. Taken together, ISNA’s accuracy is expected to be lower than that of well-developed computational models [12,13]. Despite these limitations, this is the first report of system capable of constructing a TRN “black box” solely from transcriptomic data. We anticipate that future advancements integrating ISNA with bioinformatics theories, improved learning algorithms, and refined methodologies will lead to more powerful and accurate TRN reconstruction systems.

## Supporting information

S1 Fig The digraph to visualize the relationships among core TFs at each gene body region in control HUVEC. The digraph was derived from derivative factors of the gradient descent method to visualize the relationships among core TFs at each gene body region in control HUVEC. When the TFs had a larger number of edges with the entering direction to the TFs, the TFs were situated at a more center position. Left bar and color of TFs: the number of edges with the forward direction from the TFs. Width of edges: reflecting the size of derivative factors.
(TIF)

S2 Fig The digraph to visualize the relationships among core TFs at each gene body region in EndMT of HUVEC. The digraph was derived from the rate of derivative factors of the gradient descent method (EndMT/control HUVEC values) in activated or repressed TFs in EndMT to visualize the relationships among core TFs at each gene body region. When the TFs had a larger number of edges with the entering direction to the TFs, the TFs were situated at a more center position. Left bar and color of TFs: the number of edges with the forward direction from the TFs. Width of edges: reflecting the size of derivative factors.
(TIF)

S3 Fig The digraph to visualize the relationships among core TFs at each gene body region in human endometrial epithelial cells. The digraph was derived from derivative factors of the gradient descent method to visualize the relationships among core TFs at each gene body region in human endometrial epithelial cells. When the TFs had a larger number of edges with the entering direction to the TFs, the TFs were situated at a more center position. Left bar and color of TFs: the number of edges with the forward direction from the TFs. Width of edges: reflecting the size of derivative factors.
(TIF)

S4 Fig The localization of HMGA2 in the epithelia of female reproductive tracts. The immunofluorescence images for HMGA2 (green) in the uterus, vagina, ampulla of oviduct, and isthmus of oviduct of 3-month-old intact diestrus and estrus mice, 3-month-old OVX mice with oil or E2 treatment, and mice at postnatal day 3. Blue: the nuclei. Left images: only HMGA2. Right images: merged images with HMGA2 and nuclei. White arrows: the uterine glands. Dash line: basement membrane. n = 3, biologically independent.
(TIF)

S5 Fig The localization of DLX5 in the epithelia of female reproductive tracts. The immunofluorescence images for DLX5 (green) in the uterus, vagina, ampulla of oviduct, and isthmus of oviduct of 3-month-old intact diestrus and estrus mice, 3-month-old OVX mice with oil or E2 treatment, and mice at postnatal day 3. Blue: the nuclei. Left images: only DLX5. Right images: merged images with DLX5 and nuclei. White arrows: the uterine glands. Dash line: basement membrane. n = 3, biologically independent.
(TIF)

S6 Fig The localization of HMGA2 in the epithelium of the junction between the oviduct and uterus. The immunofluorescence images for HMGA2 (green) in the epithelium of junction between oviduct and uterus of 3-month-old OVX mice with oil or E2 treatment. Blue: the nuclei. Left images: only HMGA2. Right images: merged images with HMGA2 and nuclei. n = 3, biologically independent.
(TIF)
