# Supplementary figures and images for: In silico screening system based on a transcription factors regulatory network only using transcriptomic data
Source: PLoS One. 2025 Apr 7;20(4):e0319971. doi: 10.1371/journal.pone.0319971 (PMC11975132; doi:10.1371/journal.pone.0319971)

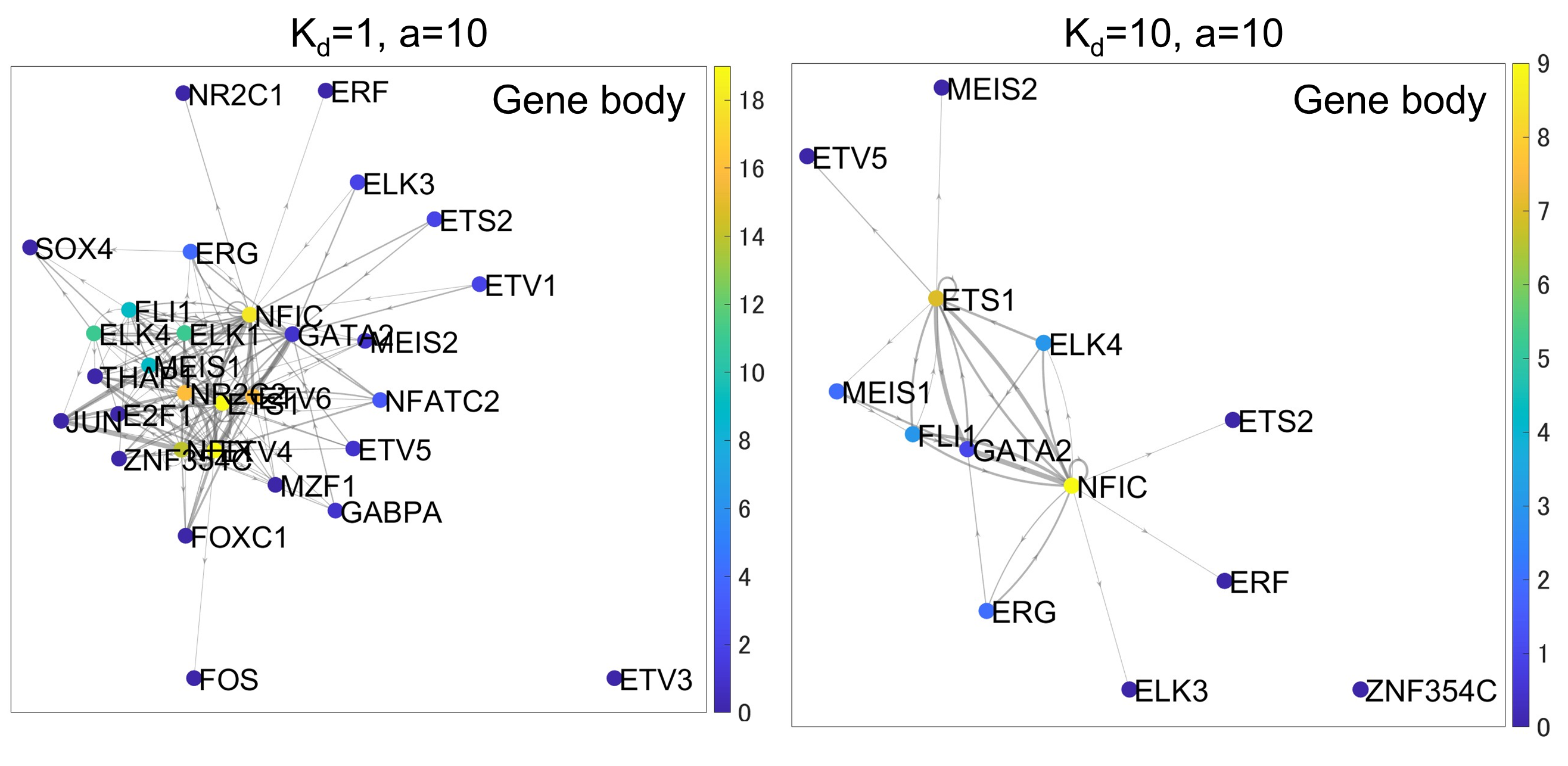

Supplement: S1 Fig — The digraph was derived from derivative factors of the gradient descent method to visualize the relationships among core TFs at each gene body region in control HUVEC. When the TFs had a larger number of edges with the entering direction to the TFs, the TFs were situated at a more center position. Left bar and color of TFs: the number of edges with the forward direction from the TFs. Width of edges: reflecting the size of derivative factors. (TIF) [file pone.0319971.s001.TIF]

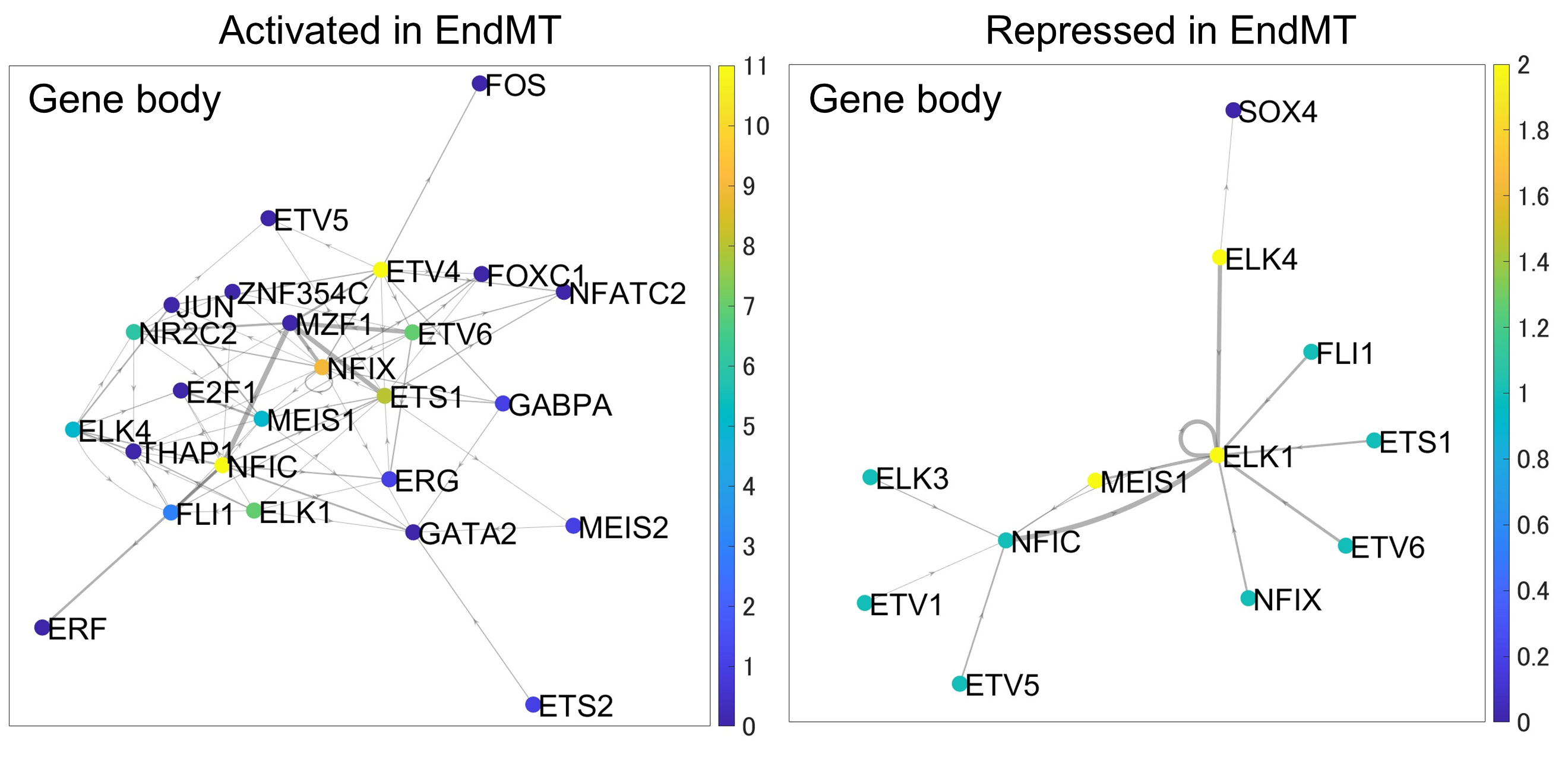

Supplement: S2 Fig — The digraph was derived from the rate of derivative factors of the gradient descent method (EndMT/control HUVEC values) in activated or repressed TFs in EndMT to visualize the relationships among core TFs at each gene body region. When the TFs had a larger number of edges with the entering direction to the TFs, the TFs were situated at a more center position. Left bar and color of TFs: the number of edges with the forward direction from the TFs. Width of edges: reflecting the size of derivative factors. (TIF) [file pone.0319971.s002.TIF]

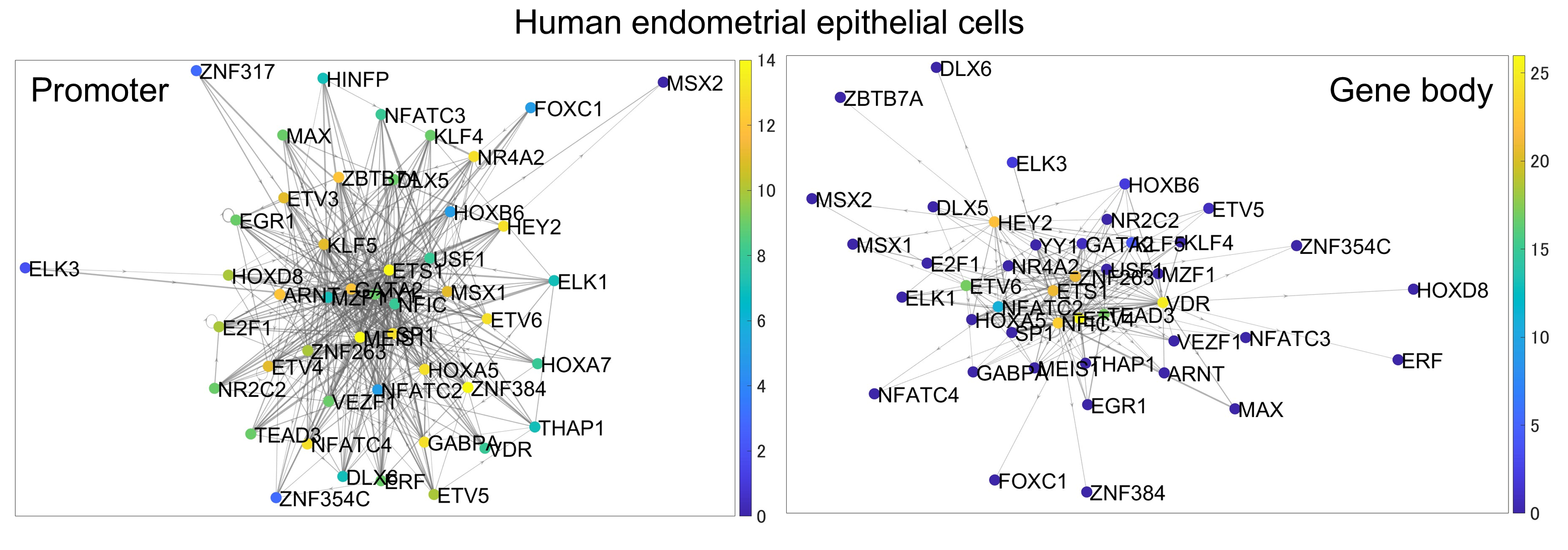

Supplement: S3 Fig — The digraph was derived from derivative factors of the gradient descent method to visualize the relationships among core TFs at each gene body region in human endometrial epithelial cells. When the TFs had a larger number of edges with the entering direction to the TFs, the TFs were situated at a more center position. Left bar and color of TFs: the number of edges with the forward direction from the TFs. Width of edges: reflecting the size of derivative factors. (TIF) [file pone.0319971.s003.TIF]

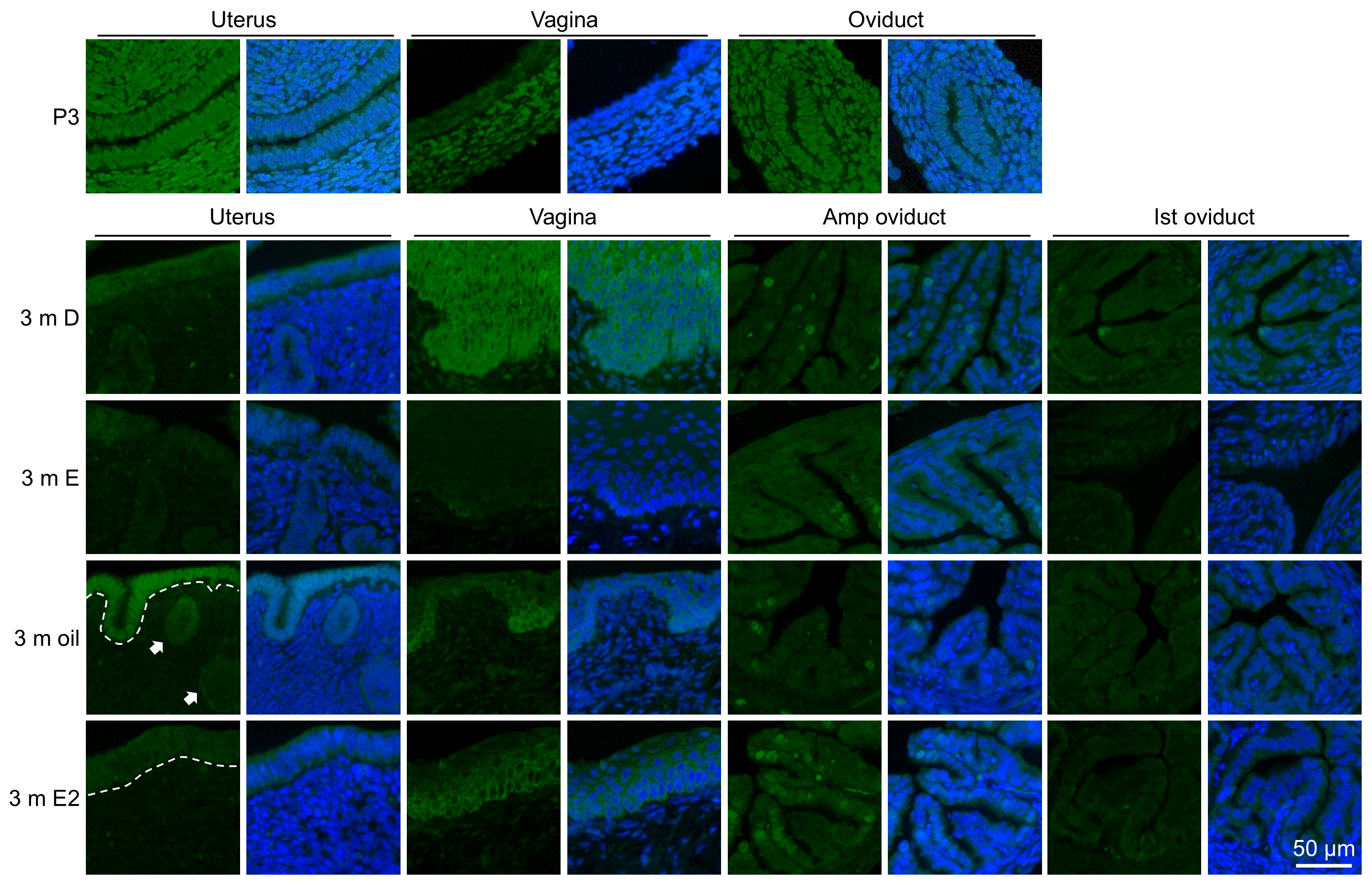

Supplement: S4 Fig — The immunofluorescence images for HMGA2 (green) in the uterus, vagina, ampulla of oviduct, and isthmus of oviduct of 3-month-old intact diestrus and estrus mice, 3-month-old OVX mice with oil or E2 treatment, and mice at postnatal day 3. Blue: the nuclei. Left images: only HMGA2. Right images: merged images with HMGA2 and nuclei. White arrows: the uterine glands. Dash line: basement membrane. n = 3, biologically independent. (TIF) [file pone.0319971.s004.TIF]

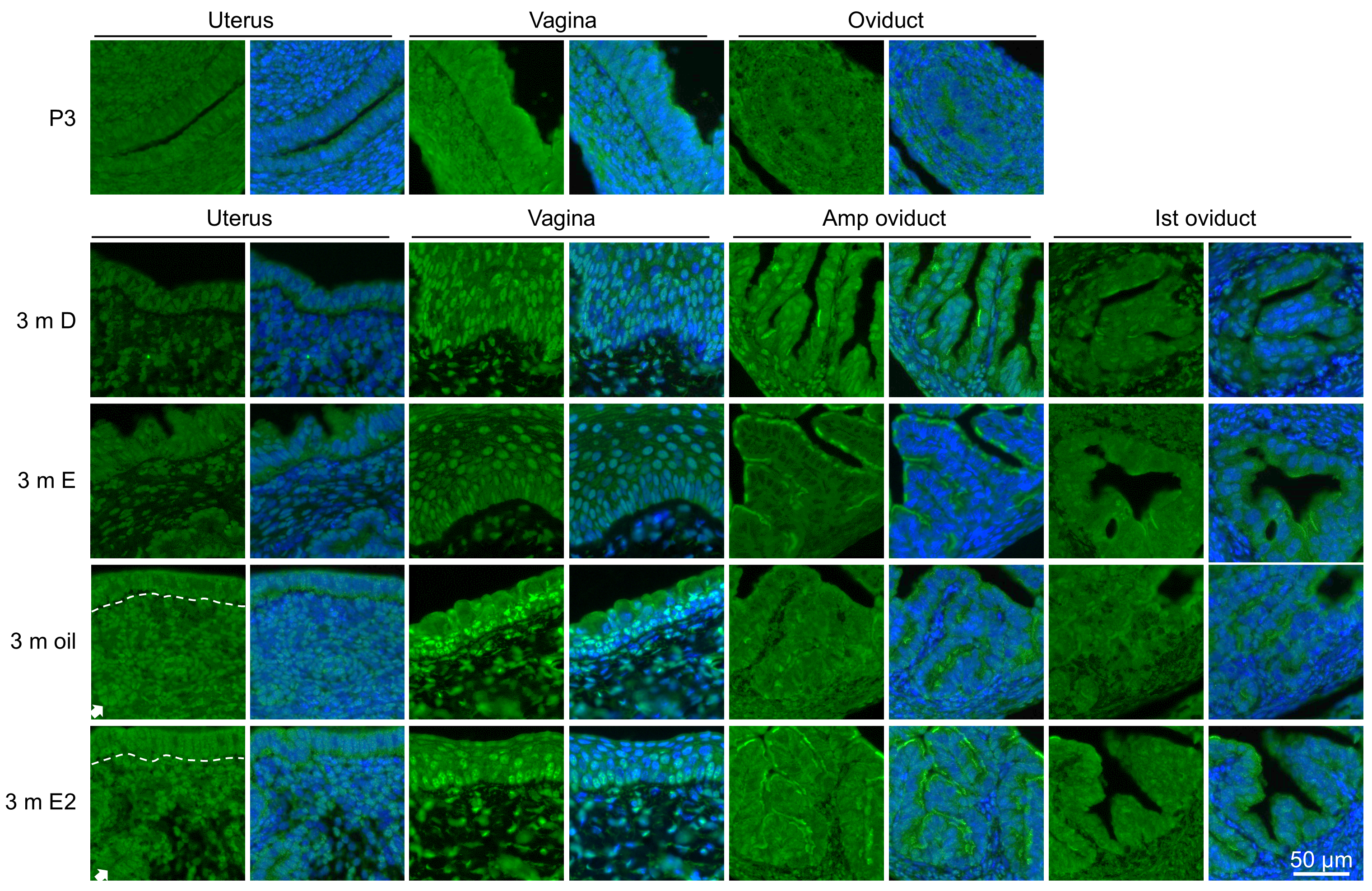

Supplement: S5 Fig — The immunofluorescence images for DLX5 (green) in the uterus, vagina, ampulla of oviduct, and isthmus of oviduct of 3-month-old intact diestrus and estrus mice, 3-month-old OVX mice with oil or E2 treatment, and mice at postnatal day 3. Blue: the nuclei. Left images: only DLX5. Right images: merged images with DLX5 and nuclei. White arrows: the uterine glands. Dash line: basement membrane. n = 3, biologically independent. (TIF) [file pone.0319971.s005.TIF]

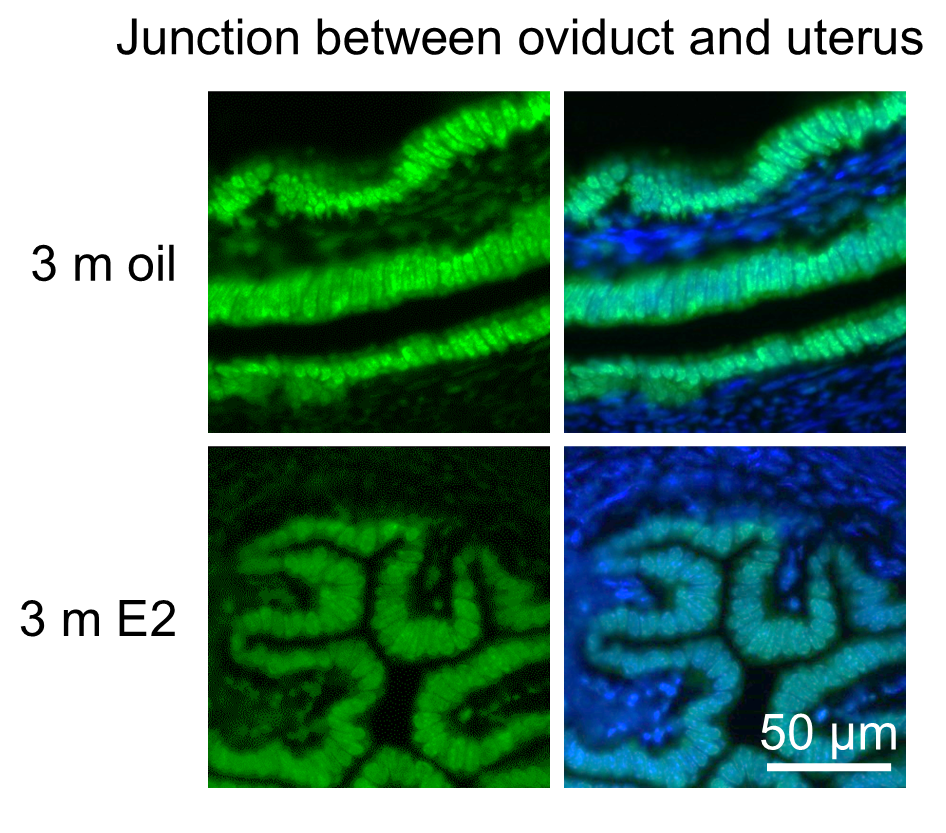

Supplement: S6 Fig — The immunofluorescence images for HMGA2 (green) in the epithelium of junction between oviduct and uterus of 3-month-old OVX mice with oil or E2 treatment. Blue: the nuclei. Left images: only HMGA2. Right images: merged images with HMGA2 and nuclei. n = 3, biologically independent. (TIF) [file pone.0319971.s006.TIF]
